# Peripheral Vascular and Physiological Responses Associated with Electroacupuncture in Chronic Nonspecific Low Back Pain: A Randomized Controlled Trial

**DOI:** 10.3390/healthcare14213792

**Published:** 2026-11-06

**Authors:** Xin Wang, Hui Liu, Qunbang Zhu, Qi Zou, Yixuan Wang, Wei Gu

**Affiliations:** 1 Naval Medical Center, Shanghai 200052, China; 13262982685@163.com (X.W.); 2221517015@sus.edu.cn (H.L.); zhuqunbang19871015@126.com (Q.Z.); 17852137782@163.com (Q.Z.); 22210290021@m.fudan.edu.cn (Y.W.); 2 Department of Traditional Chinese Medicine, Naval Medical University ( Second Military Medical University), Shanghai 200433, China

**Keywords:** electroacupuncture, chronic nonspecific low back pain, microcirculation, skin temperature, objective physiological measurement

## Abstract

**Highlights:**

**What are the main findings?**

• Electroacupuncture produced clinically meaningful improvements in pain and disability in patients with chronic nonspecific low back pain after a two-week treatment course.
• Electroacupuncture induced measurable local physiological responses, particularly increased microcirculatory perfusion, which was associated with pain reduction.

**What are the implications of the main findings?**

• Local blood flow changes may serve as a potential objective physiological correlate for evaluating acupuncture-related symptom improvement.
• Combining clinical outcomes with noninvasive peripheral physiological measurements may provide a new approach for investigating acupuncture mechanisms.

**Abstract:**

**Objective:** This exploratory study aimed to evaluate the effects of electroacupuncture (EA) on pain and functional outcomes in patients with chronic nonspecific low back pain (CNLBP), and to investigate objective peripheral physiological responses associated with clinical improvement. **Methods:** In this prospective, double-blind, randomized sham-controlled trial, adults with CNLBP received EA at Shenshu (BL23) and an ipsilateral Ashi acupoint three times weekly for 2 weeks, followed by a 4-week follow-up. The primary outcome was pain intensity assessed by the Numeric Rating Scale (NRS), with responder analysis based on the minimal clinically important difference (MCID; ΔNRS ≥ 2). Secondary outcomes included the Oswestry Disability Index (ODI), local microcirculatory perfusion, skin temperature, and muscle tissue properties. **Results:** Compared with sham EA, EA significantly reduced pain intensity after the first session, after six sessions, and at follow-up (*p* < 0.003; *r* = 0.70–0.84). MCID responder rates were significantly higher in the EA group after the first session, six sessions and at follow-up (*p* < 0.003; RR = 3.75 and 6.00 after six sessions and follow-up, respectively). EA increased local blood flow at BL23 after the first and sixth sessions. Skin temperature showed only a transient within-group increase, without a significant adjusted between-group difference. Similar trends were observed at Ashi acupoint, and increased blood flow was associated with pain improvement. **Conclusions:** EA improved pain and functional outcomes in patients with CNLBP while inducing measurable peripheral vascular and physiological responses. Increased local blood flow may represent a candidate physiological correlate of EA-related symptom improvement. These findings support the exploration of objective physiological markers in acupuncture research.

## 1. Introduction

Acupuncture and electroacupuncture (EA) have been widely applied for musculoskeletal pain [1,2]. Although patients often report subjective improvements in pain relief and functional recovery, efficacy assessment still depends largely on self-reported measures such as pain scores and functional scales [3]. At the clinical trial level, limited differences exist between EA and sham electroacupuncture (SA), leading to situations where patients report improvement but objective measures show insufficient change [4]. Therefore, objective and reproducible measures that can characterize local tissue responses during acupuncture treatment warrant further investigation.

From the perspective of mechanism of action, the therapeutic effect of acupuncture (including EA) is initiated by physical stimulation of local muscles [5]. Needle insertion and manipulation induce mechanical deformation of local tissues, triggering a cascade of biochemical and neuroimmune reactions [6,7,8,9]. In acupuncture analgesia, this pathway is most clearly illustrated, and it results in therapeutic effects at the peripheral-to-central level [10]. In this process, the immediate quantifiable biological phenomena resulting from mechanical stimulation of acupuncture in peripheral tissues are changes in local blood flow, tissue temperature, and physical properties [11,12]. These parameters are not only noninvasive and reproducible, but also include real-time information on the microcirculation, tissue perfusion, and local metabolic status at the acupuncture site and change dynamically during treatment [7,13,14]. Compared with invasive inflammatory biomarkers [15,16], these objective physiological measures may offer a practical approach for monitoring acupuncture-induced peripheral responses and evaluating therapeutic effects based on biological mechanisms.

Chronic nonspecific low back pain (CNLBP) is defined as low back pain that does not have a specific pathology and persists over a long period of time [17]. It has high prevalence [18] and disability rates, and makes a significant contribution to the global disease burden [19,20,21]. Clinical guidelines and evidence-based recommendations have recognized acupuncture as a potential non-pharmacological treatment option for CNLBP [22,23]. EA, which combines acupuncture stimulation with electrical stimulation, has also been investigated in patients with CNLBP and may provide additional physiological stimulation compared with manual acupuncture [4,24]. However, the difference in efficacy for EA is disputed [25]. One important limitation is the lack of objective biomarkers capable of reflecting peripheral tissue responses associated with acupuncture stimulation.

Thus, using CNLBP as the disease model, the main goal of this study was to determine the clinical effect of EA compared with sham acupuncture. In addition, we aimed to characterize EA-induced local vascular and physiological responses by monitoring changes in local blood flow, tissue temperature, and muscle physical properties before and after acupuncture, and to investigate the relationship of these changes with the improvement in clinical symptoms. The results of this study could offer preliminary objective evidence to assess physiological responses related to acupuncture and contribute to a better understanding of vascular and tissue adaptations underlying EA effects.

## 2. Methods

### 2.1. Study Design and Participants

A prospective, randomized, double-blind, sham-controlled trial was undertaken at Changhai Hospital, Shanghai. Approval for the research protocol came from the hospital’s ethics committee (No. CHEC2023-191), and all participants gave written informed consent beforehand. All participants were allowed to continue their usual care. The sham procedure was non-invasive and low-risk. After the 4-week follow-up, participants in the sham group were offered active EA free of charge. The study had been registered in advance with the Chinese Clinical Trial Registry (ChiCTR2300075954).

Participants were recruited from outpatient acupuncture clinics. Inclusion criteria were as follows: a diagnosis of CNLBP consistent with international guidelines [26]; predominantly left-sided low back pain; male participants aged 18–40 years; symptom duration ≥ 6 months; numeric rating scale (NRS) score ≥ 3; and body mass index (BMI) 18.5–23.9 kg/m^2^. Exclusion criteria comprised prior lumbar surgery; radiculopathy or specific spinal pathology; severe cardiovascular, hepatic, or renal disease; recent acupuncture or other interventions for low back pain within 8 weeks; implanted electronic devices; severe psychiatric disorders.

The eligibility criteria were selected to reduce heterogeneity in the physiological measurements. The age and BMI criteria were chosen to minimize the potential influence of age-related vascular changes, obesity-related tissue mechanical alterations, and metabolic factors on peripheral physiological measurements. Male sex was required to avoid potential confounding by menstrual-cycle-related fluctuations in peripheral microcirculation and skin temperature. The restriction to predominantly left-sided symptoms was applied to standardize measurement sites and acupuncture procedures, thereby reducing variability associated with bilateral symptom distribution.

### 2.2. Randomization and Blinding

After baseline questionnaires were completed, we randomly allocated those who met the eligibility criteria in a 1:1 ratio to either the EA or the SA group via a centralized online randomization system. The random sequence was generated by an independent statistician using the R software (version 4.2.0). Participants and outcome assessors, including those responsible for physiological measurements and data processing, were blinded to group allocation until the study was completed. The acupuncturists had no access to the results of the assessment or to data entry to avoid performance and detection bias. Blinding was assessed at the final treatment session using a standardized blinding questionnaire that asked participants to indicate if they thought they had received EA, SA, or were uncertain.

### 2.3. Interventions

All treatment sessions were performed by a licensed acupuncturist who had received undergraduate training in acupuncture, and had clinically practiced for over five years. Before the study started, the acupuncturist was systematically trained on the study protocol to ensure consistency of treatment procedures. Shenshu (BL23) was selected as a representative classical lumbar acupoint frequently used in LBP treatment [27], and the ipsilateral Ashi acupoint was selected as an individualized pain-sensitive site [28] (Figure S1). This combination was intended to reflect commonly used clinical acupuncture practice while allowing assessment of local physiological responses. Patients in the EA group received EA at the left BL23 and the ipsilateral Ashi acupoint. Manual manipulation of the needles was applied for at least 10 s to elicit the Deqi sensation [3], characterized by soreness, numbness, distension, or heaviness. EA was performed at Shenshu (BL23) and the Ashi acupoint using disposable stainless-steel needles (Huazhen, JiaJian brand, 0.30 × 40 mm, produced by JiaJian Medical Device Co., Ltd., Wuxi, Jiangsu, China). Subsequently, the EA apparatus (KWD-808I Pulse Acupuncture Therapy Device, Yingdi brand, 220 V, 50 Hz, produced by Changzhou Electronic Medical Device Co., Ltd., Changzhou, China) was connected to the needles using a 2/100 Hz sparse-dense wave [29,30]. The needles were retained for 30 min, and the intensity was adjusted according to each participant’s maximal tolerance. Treatments comprised 6 sessions over 2 weeks (three per week), with a 4-week post-treatment follow-up [31]. Hand hygiene was performed before each session. The skin at each acupoint was cleaned with 75% alcohol swabs and allowed to dry before needle insertion. After removal, each needle was immediately discarded into a sharps container, and no needle was reused.

Patients randomized to the SA group received nonpenetrating sham acupuncture at Shenshu (BL23) and the Ashi acupoint. A tube cannula was positioned and fixed at Shenshu (BL23) and the Ashi acupoint, and a disposable, overlapping blunt-tip needle was applied. Once the blunt tip contacted the skin, it rebounded into the needle handle (1.3 cm), and no manual manipulation was performed [25]. The paired electrodes of the same EA device were connected to the sham needles. The device remained switched on with the indicator light flashing, but the output current was set to 0 mA [32]. No needle penetration was performed and no electrical current was delivered in the SA group. Participants were told that they might or might not feel a mild electrical sensation [33]. The treatment schedule, session duration, and weekly treatment frequency were identical between the EA and SA groups to minimize procedural differences and facilitate participant blinding.

### 2.4. Outcome Measures

#### 2.4.1. Primary Outcome

The primary outcome was pain intensity assessed using NRS. Minimal clinically important difference (MCID) was used for responder analysis [34]. Assessment time points included baseline, after the first treatment session, at 14 days (after six sessions), and at the 4-week post-treatment follow-up. A responder was defined as a participant achieving an absolute reduction of ≥ 2 points in NRS from baseline. This threshold was selected based on previous studies suggesting that a 2-point reduction on the NRS represents a clinically meaningful improvement in pain intensity at the individual patient level [35]. Although not explicitly prespecified in the original protocol, it was defined in the statistical analysis plan before data analysis.

#### 2.4.2. Secondary Outcomes

The secondary outcomes included functional disability using the Oswestry Disability Index (ODI) [36], local microcirculatory perfusion, skin temperature, and tissue mechanical properties, including tone, stiffness, and elasticity. Peripheral physiological responses in local tissues were measured at the primary acupoint Shenshu (BL23) and the exploratory Ashi point, defined as the L5/S1 pain-sensitive point identified by palpation. At baseline, immediately after needle removal following the first treatment session, and after the six-session treatment course, all secondary outcome measures were evaluated.

All physiological measurements were performed in a dedicated treatment room under controlled environmental conditions (temperature: 25 ± 1 °C; relative humidity: 50 ± 10%). Before measurements, participants were allowed to rest in the examination room for approximately 10 min to adapt to the environmental conditions. During assessment, doors and windows were closed, curtains were drawn, and stable lighting conditions were maintained. Participants were positioned prone, informed about the measurement procedures, and instructed to remain relaxed and still during data acquisition. All devices were checked according to the manufacturers’ instructions before data collection. Measurement sites were identified by palpation and marked before assessment. Predefined regions of interest (ROIs) were placed around each marked acupoint (BL23 and Ashi) and applied consistently across participants and time points. For laser speckle contrast imaging, a circular ROI of 1 cm^2^ was placed around each marked acupoint. For infrared thermography, a rectangular ROI of 1 × 1 cm was placed around the same acupoints. The order of physiological measurements was standardized across all participants. At each post-treatment assessment, laser speckle contrast imaging and infrared thermography were started before needle removal and continued for 30 s after needle removal. MyotonPRO measurements were performed immediately after completion of these recordings. The entire post-treatment measurement sequence was completed within 1 min after needle removal. This sequence and timing were kept consistent across participants for both post-treatment assessments.

Local microcirculatory perfusion was assessed using a laser speckle contrast imaging system (RFSLI ZW/RFLSI III, RWD Life Science Co., Ltd., Shenzhen, China), which provides a non-invasive and reproducible assessment of regional superficial blood perfusion [37]. The imaging device was positioned approximately 250 mm above the measurement area and manually focused. Continuous recording was performed for 30 s using a 5 × 5 cm scanning window, high-resolution mode (0.1 mm), and a sampling frequency of 20 images/s. Within each 30 s recording, a stable perfusion segment of 10 s was selected based on the absence of motion artifacts and a stable signal, and the mean perfusion value of this segment was calculated for each ROI.

Skin temperature was assessed using an infrared thermography camera (FLIR T630SC, FLIR Systems, Inc., Wilsonville, OR, USA), which enables objective and non-contact evaluation of regional temperature distribution [38]. The camera was positioned at a fixed distance of 80 cm from the measurement area, perpendicular to the skin surface, and the emissivity was set to 0.98. Imaging was performed after ensuring that the skin surface was dry and free from direct airflow. After image acquisition, three consecutive frames were selected and averaged to obtain the representative temperature for each ROI.

Tissue mechanical properties were assessed using MyotonPRO (Myoton AS, Tallinn, Estonia), which has demonstrated acceptable reliability for quantifying superficial tissue mechanical properties, including tone, stiffness, and elasticity [39,40]. The probe was placed perpendicular to the skin surface at the marked measurement sites. Three consecutive measurements were performed at each site, and the mean value was used for analysis. The impulse duration was 15 ms, with an interval of 0.8 s and a delay time of 0.7 s. The measured parameters included natural oscillation frequency (F), dynamic stiffness (S), and logarithmic decrement (D). A recording was accepted when the device successfully completed the measurement without obvious movement or technical interference. Measurements were repeated if the recording was unstable, interrupted, or if the coefficient of variation among the three consecutive measurements exceeded 10%.

### 2.5. Study Power

The sample size was determined according to the primary outcome, NRS pain intensity. Based on a pilot study involving 10 participants per group (total 20), the estimated between-group difference in NRS change from baseline after the 2-week intervention was 1.4 points, with a pooled standard deviation of 1.6. None of the pilot participants were included in the present trial. The sample size calculation was performed using G*Power 3.1 with a two-sided alpha level of 0.05 and 90% power, resulting in a requirement of 27 participants per group. Considering an anticipated dropout rate of 10–15%, we planned to enroll 30 participants per group (total 60). The study was powered specifically for the primary comparison of NRS change from baseline after completion of the six-session treatment course; other NRS time points were analyzed as secondary assessments.

### 2.6. Statistical Analysis

SPSS 20.0 (IBM Corp., Armonk, NY, USA) was used for statistical analysis. Baseline characteristics were summarized for all participants in the randomized groups. Baseline between-group comparisons were performed for descriptive purposes only. *p* values are reported as descriptive summaries and should not be interpreted as formal hypothesis tests. Normality of the continuous variables was tested by the Shapiro–Wilk test. Normally distributed variables were presented as mean ± SD, with independent-samples *t* tests used for group contrasts; non-normal variables were shown as medians (IQR), with group comparisons based on the Mann–Whitney U test. For categorical data, we applied Fisher’s exact or chi-square tests when indicated.

Nonparametric procedures were employed in the analysis of all outcome variables (NRS, ODI, local microcirculatory perfusion, skin temperature, and tissue mechanical parameters) as they were not normally distributed. Intra-group comparisons between the baseline and post-treatment evaluations were carried out using the Wilcoxon signed-rank test, while the Mann–Whitney U test was applied on the Δ values (post-treatment minus baseline). Bonferroni correction was used to account for the greater risk of a Type I error for outcomes requiring multiple post-baseline comparisons. Raw *p* values and Bonferroni-adjusted *p* values have been shown in the respective tables, and statistical significance has been calculated using the Bonferroni-adjusted *p* value unless otherwise stated. The multiple-comparison adjustment did not apply to the baseline between-group comparisons, which were descriptive. Effect size was computed as r = |Z|/$\sqrt{N}$, where Z was the value obtained from the Wilcoxon signed-rank test or Mann–Whitney U test. For within-group comparisons, N represented the number of paired observations, whereas for between-group comparisons, N represented the total number of participants. According to Cohen’s criteria, r values of 0.10, 0.30, and 0.50 correspond to small, medium, and large effect sizes, respectively.

All clinical and physiological outcomes were assessed in the per-protocol/available-case dataset of participants who had complete post-baseline measurements (*n* = 59). To evaluate the effect of attrition on clinical outcomes, additional ITT sensitivity analyses were performed for NRS scores, MCID responder status, and ODI, with missing values for the withdrawn participant imputed using baseline observation carried forward. The ITT results were in agreement with the complete-case analysis (Supplementary Tables S7–S9), indicating that attrition did not materially affect the clinical findings. For physiological parameters, missing data were not imputed because reliable imputation requires adequate signal quality and complete recordings. For the MCID responder analysis, clinically significant improvement was defined as a reduction of ≥ 2 points in NRS from baseline. Between-group differences in responder proportions at different post-baseline time points were assessed using the χ^2^ test or Fisher’s exact test, as appropriate, and risk ratios with 95% confidence intervals were calculated.

For the blinding assessment, the distribution of participants’ treatment guesses between the EA and SA groups was compared using the χ^2^ test. No blinding index was prespecified in the trial protocol, and blinding results were therefore summarized descriptively. To explore the association between local physiological changes and clinical improvement, Spearman’s rank correlation analyses were performed between change-from-baseline values of local blood flow and skin temperature at BL23 and changes in NRS and ODI scores. All participants with complete paired data were included in this exploratory analysis. Spearman’s ρ coefficients are used to report correlations, with 95% confidence intervals computed from Fisher’s z transformation. For exploratory correlation analyses, nominal two-sided *p* values were reported without additional multiplicity correction. These findings were interpreted cautiously as hypothesis-generating observations. All other statistical tests used a two-sided approach, and the threshold for significance was set at adjusted *p* < 0.05.

### 2.7. Adverse Events

Adverse events were monitored throughout the intervention period through systematic inquiry after each treatment session and spontaneous reporting. Participants were routinely asked about any discomfort or adverse symptoms, and all reported events were recorded by the study staff.

## 3. Results

### 3.1. Trial Flow

**Figure 1:**
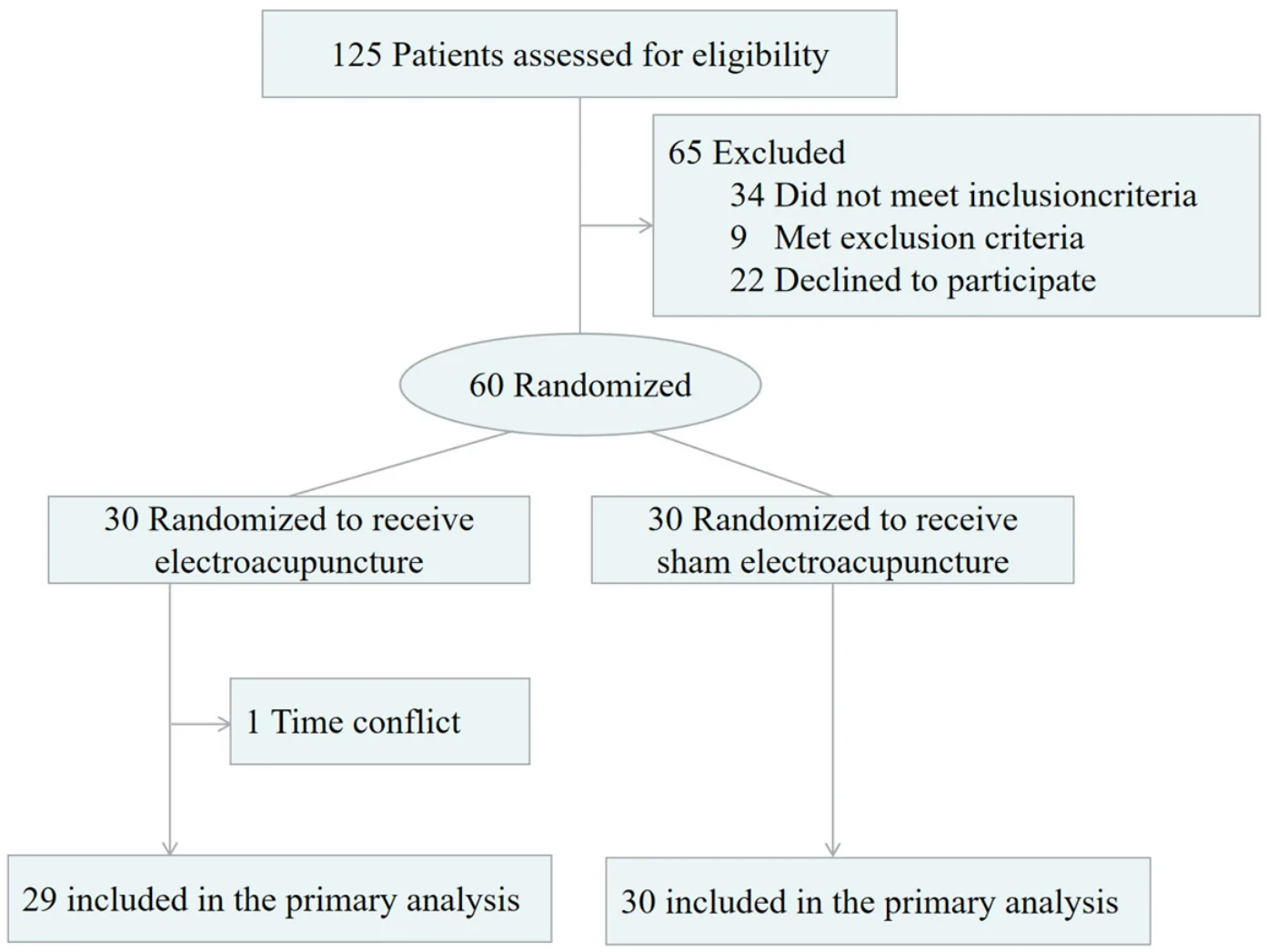
Flow Diagram of the Participants. EA: electroacupuncture; SA: sham electroacupuncture.

A total of 125 patients were assessed for eligibility (Figure 1). Following screening, 65 participants were excluded: 34 failed to meet inclusion criteria, 9 met exclusion criteria, and 22 declined participation. A total of 60 participants were randomized to the EA group (n = 30) and the SA group (n = 30). One participant in the EA group withdrew because of scheduling conflicts. Therefore, 29 participants in the EA group and all 30 participants in the SA group completed the treatment course and were included in the complete-case analyses. Baseline demographic and clinical characteristics were comparable between groups and are summarized descriptively in Table 1 and Table S10. The blinding assessment showed that 18 of 29 participants in the EA group guessed EA, 7 guessed SA, and 4 were uncertain. In the SA group, 11 of 30 participants guessed EA, 9 guessed SA, and 10 were uncertain. There was no significant between-group difference in the distribution of treatment guesses (χ^2^ = 4.50, *p* = 0.11).

### 3.2. Pain Intensity and Responder Analysis

**Figure 2:**
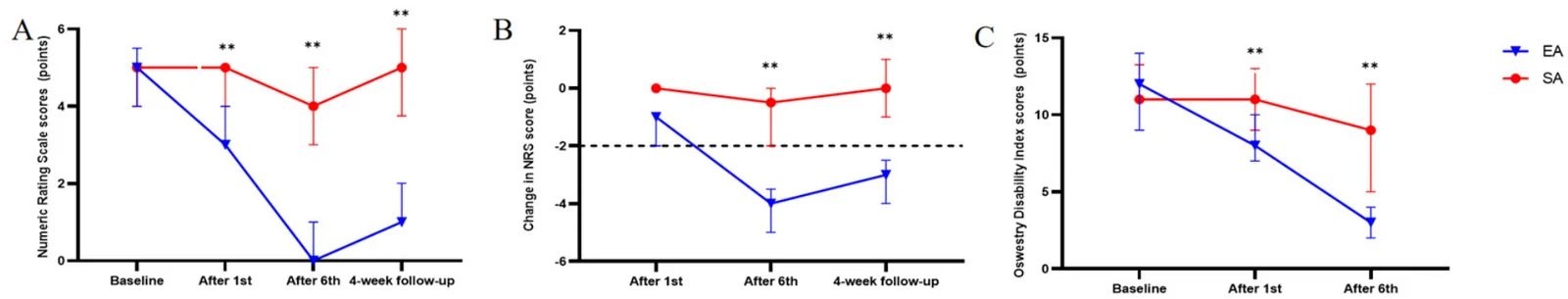
Clinical effects of electroacupuncture on pain and functional outcomes. (**A**) Changes in the Numeric Rating Scale (NRS) scores over time in the electroacupuncture (EA) and sham acupuncture (SA) groups. (**B**) Changes in NRS scores from baseline in the EA and SA groups. The dashed line indicates the MCID threshold (≥2-point reduction in NRS). (**C**) Changes in Oswestry Disability Index (ODI) scores during the intervention period. Data are presented as median with interquartile range (IQR). *p* values were adjusted using Bonferroni correction where applicable, ** *p* < 0.01. EA: electroacupuncture; SA: sham acupuncture; NRS: Numeric Rating Scale; MCID: minimal clinically important difference; ODI: Oswestry Disability Index; IQR:interquartile range.

Compared to the SA group, the EA group showed a substantial decrease in NRS-assessed pain intensity (Table 2 and Table S11 and Figure 2A). At baseline, the median NRS scores were 5.00 (4.00, 5.00) in the EA group and 5.00 (4.00, 5.00) in the SA group. After the first treatment session, NRS scores in the EA group decreased significantly compared with the pre-treatment period (*r* = 0.89, *p* < 0.003) and no significant difference (*r* = 0.32, *p* = 0.24) was found for the SA group. A statistically significant (*r* = 0.84, *p* < 0.003) difference in change scores was found between the groups. The EA group had a significantly greater decrease in NRS scores than the SA group after the completion of the six-session protocol (*r* = 0.78, *p* < 0.003). There was a weak rebound of pain intensity in the EA group at 4 weeks post-treatment follow-up, but this was still below the baseline, while no difference from baseline was seen in the SA group. A significant between-group difference persisted at follow-up (*r* = 0.78, *p* < 0.003). Responder analysis according to the MCID showed that more than one-third of patients in the EA group experienced clinically significant improvements (11/29, 37.93%) after the first treatment session. No patients in the sham group reached this threshold (Table 3 and Figure 2B). The between-group difference was statistically significant (*p* < 0.003). After completion of the six-session treatment protocol, the proportion of responders increased markedly in the EA group, with 29 of 29 patients (100.00%) achieving the MCID, compared with 8 of 30 patients (26.67%) in the sham group. The observed difference was significant, with a significantly higher probability of achieving clinically meaningful pain relief in the EA group (RR = 3.75, 95% CI: 2.07–6.79; *p* < 0.003). At the 4-week post-treatment follow-up, all patients in the EA group continued to meet the MCID criterion (29/29, 100.00%), whereas only 5 of 30 patients (16.67%) in the sham group showed clinically meaningful improvement. The between-group difference remained statistically significant (RR = 6.00, 95% CI: 2.70–13.35; *p* < 0.003). A sensitivity analysis based on the ITT principle was performed for the clinical outcome (NRS and MCID) using baseline observation carried forward for the one participant who withdrew. The ITT results were consistent with the complete-case analysis, supporting the robustness of the clinical findings (Tables S7 and S8).

### 3.3. Functional Disability

Functional disability, assessed by the ODI, improved significantly in the EA group following treatment (Table 4 and Table S12 and Figure 2C). At baseline, the median ODI scores were 12.00 (9.00, 14.00) in the EA group and 11.00 (11.00, 13.00) in the SA group. Moreover, both after the first treatment and after completion of the full treatment course, between-group comparisons of change-from-baseline ODI scores revealed significantly greater improvements in the EA group than in the SA group (After 1st: *r* = 0.72, *p* < 0.002; After 6th: *r* = 0.64, *p* < 0.002). Similarly, the ITT sensitivity analysis for ODI, using the same baseline observation carried forward approach, yielded results consistent with the complete-case analysis, confirming that the withdrawal did not materially affect the functional disability findings (Table S9).

### 3.4. Local Microcirculatory Responses

**Figure 3:**
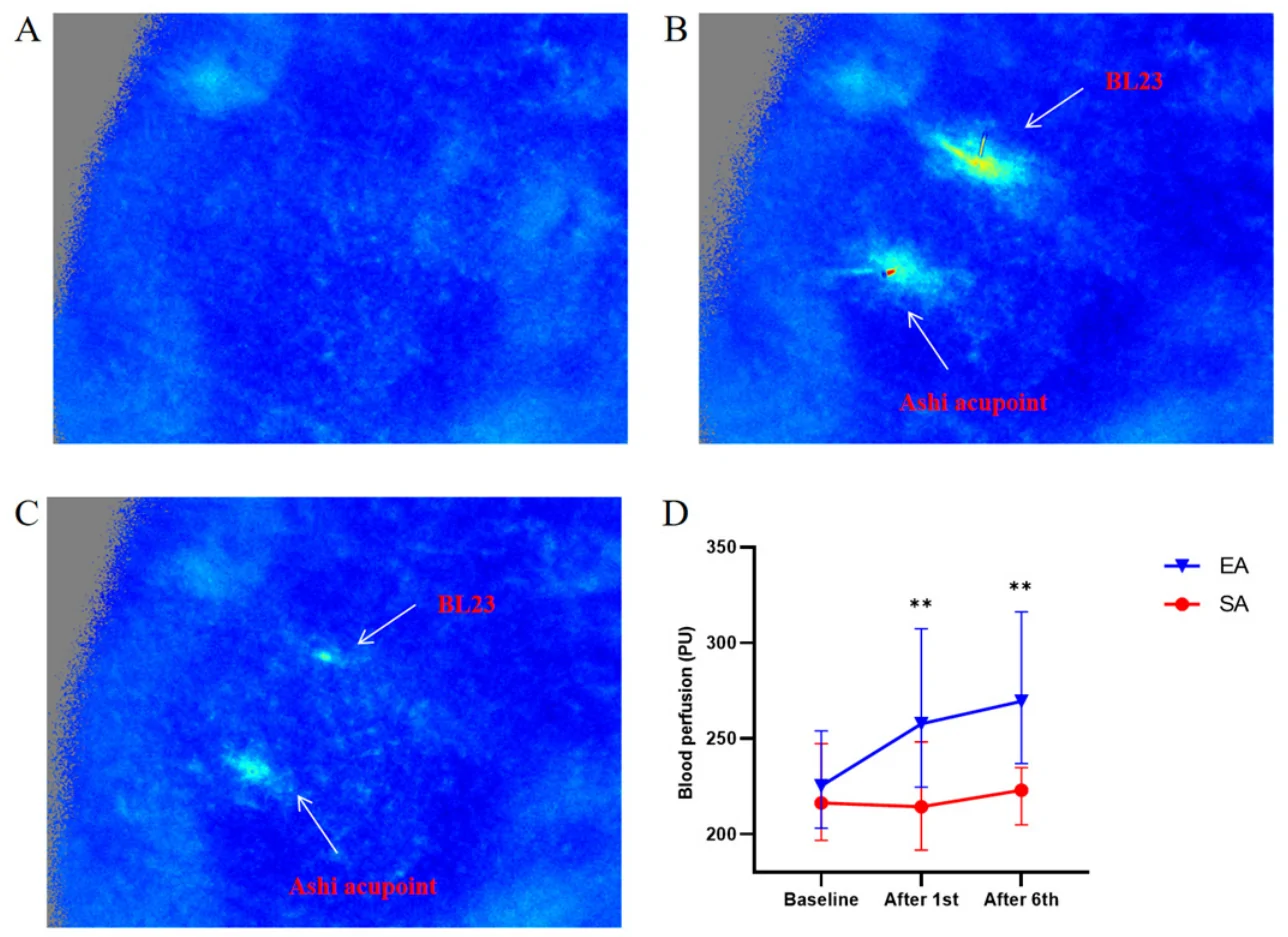
Changes in local blood perfusion following electroacupuncture treatment. (**A**–**C**) Representative laser speckle contrast imaging (LSCI) images showing dynamic changes in local blood perfusion at different stages of acupuncture stimulation: (**A**) baseline before acupuncture, (**B**) immediately after needle insertion, and (**C**) after needle removal. The locations of Shenshu (BL23) and the Ashi acupoint are indicated. (**D**) Quantitative analysis of local blood perfusion changes in the EA and SA groups at baseline, after the first treatment session, and after the sixth treatment session. Data are presented as median with interquartile range (IQR). *p* values were adjusted using Bonferroni correction where applicable, ** *p* < 0.01. EA: electroacupuncture; SA: sham acupuncture; LSCI: laser speckle contrast imaging; PU: perfusion units. IQR: interquartile range.

At baseline, the median local microcirculatory perfusion values were 225.32 (205.17, 253.65) in the EA group and 216.40 (197.56, 247.30) in the SA group (Table 5 and Table S1 and Figure 3). Within-group analysis showed that local microcirculatory perfusion in the EA group increased significantly after the first treatment and remained elevated after completion of the full treatment course (After 1st: *r* = 0.68, *p* < 0.002; After 6th: *r* = 0.64, *p* < 0.002). In contrast, we observed no significant changes in the SA group at either time point. Between-group comparisons demonstrated significantly higher microcirculatory perfusion in the EA group than in the SA group after the first treatment and after completion of all treatments (After 1st: *r* = 0.37, *p* = 0.008; After 6th: *r* = 0.40, *p* = 0.004). Similar directional changes in microcirculatory perfusion were observed at the ipsilateral Ashi acupoint across time points (Tables S1 and S2).

### 3.5. Local Thermoregulatory Responses

**Figure 4:**
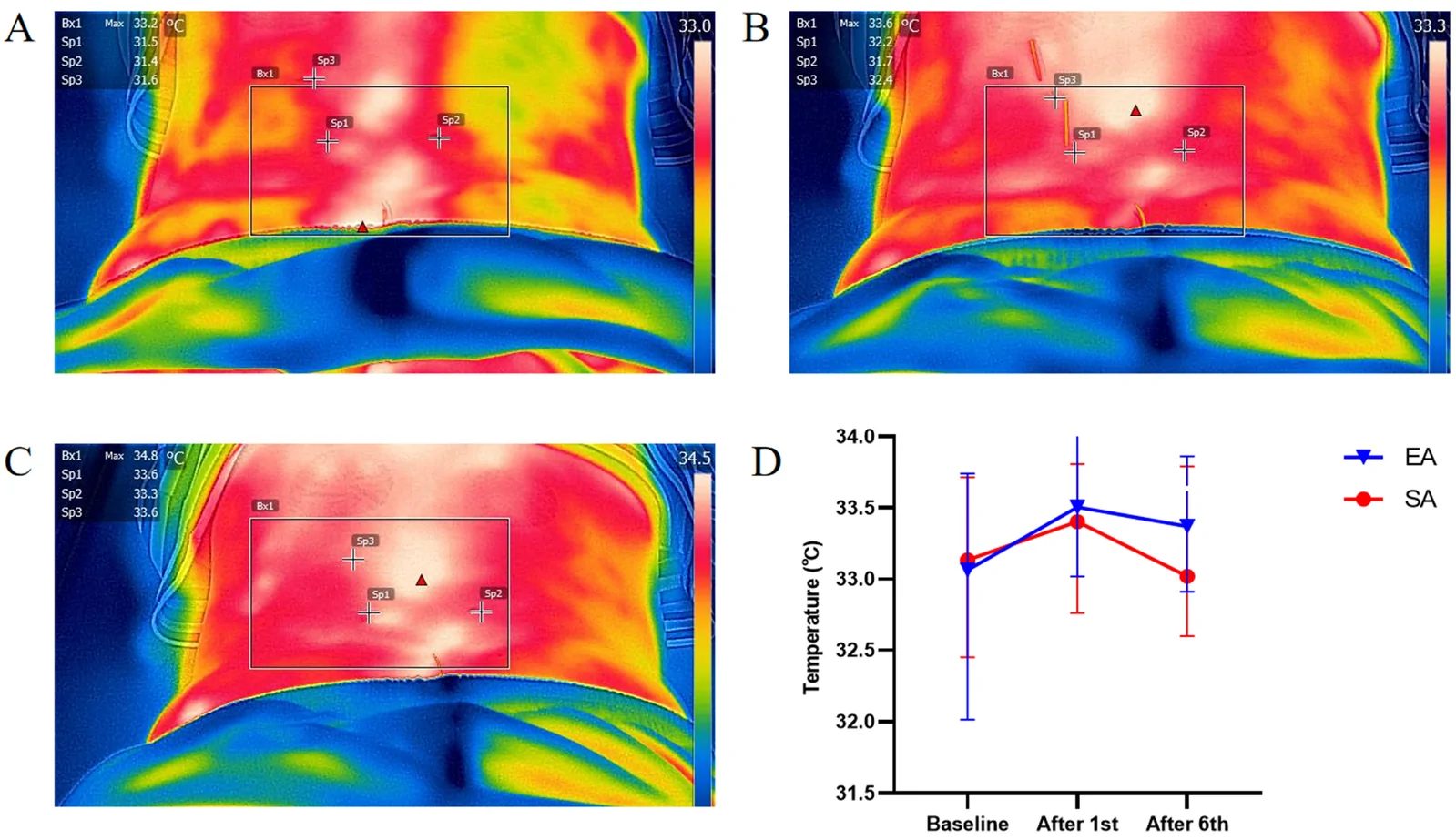
Changes in local skin temperature following electroacupuncture treatment. (**A**–**C**) Representative infrared thermographic images showing dynamic changes in local skin temperature at different stages of acupuncture stimulation: (**A**) Baseline before acupuncture, (**B**) immediately after needle insertion, and (**C**) after needle removal. The regions of interest (ROIs) are indicated. SP1 corresponds to the Ashi acupoint, whereas SP3 corresponds to the Shenshu acupoint (BL23). (**D**) Quantitative analysis of skin temperature changes in the EA and SA groups at baseline, after the first treatment session, and after the sixth treatment session. Data are presented as median with interquartile range (IQR). EA, electroacupuncture; SA, sham acupuncture; ROI, region of interest.

At baseline, the median local skin temperature values were 33.06 (32.10, 33.65) in the EA group and 33.13 (32.50, 33.71) in the SA group (Table 6 and Table S3 and Figure 4). In the within-group analysis, skin temperature in the EA group increased significantly after the first treatment compared with baseline (*r* = 0.68, *p* < 0.002) and exhibited an upward trend following completion of the full treatment course, whereas no significant changes were observed in the SA group at either time point. Skin temperature showed a marginal between-group difference after the first treatment, with higher values observed in the EA group than in the SA group. However, this difference did not remain significant after Bonferroni adjustment (*r* = 0.26, raw *p* = 0.05; adjusted *p* = 0.10). No significant between-group difference was observed after completion of all treatments (*r* = 0.17, *p* = 0.40). Skin temperature at the Ashi acupoint exhibited changes in the same direction as those observed at BL23 (Tables S3 and S4).

### 3.6. Tissue Mechanical Properties

At baseline, the MyotonPRO-derived tissue mechanical properties at the Shenshu (BL23) acupoint are summarized descriptively for the EA and SA groups (Table 7 and Table S5). No significant between-group differences were detected after the first treatment, or after the sixth treatment across any of the assessed biomechanical parameters (all *p* > 0.46). Tissue mechanical parameters assessed at the Ashi acupoint showed a similar lack of significant between-group differences across time points (Tables S5 and S6).

### 3.7. Association Between Local Physiological Responses and Clinical Improvement

Exploratory Spearman correlation analyses showed that greater increases in local microcirculatory perfusion at BL23 were associated with larger reductions in NRS scores (ρ = −0.34, 95% CI: −0.55 to −0.09, *p* = 0.008; Table 8). However, the association between changes in local blood flow and ODI scores was not statistically significant (ρ = −0.20, 95% CI: −0.43 to 0.06, *p* = 0.14). Changes in skin temperature at BL23 showed a nonsignificant trend toward association with reductions in NRS scores (ρ = −0.23, 95% CI: −0.46 to 0.03, *p* = 0.08) and a weak association with reductions in ODI scores (ρ = −0.27, 95% CI: −0.49 to −0.01, *p* = 0.04). Because these correlation analyses were exploratory, the reported *p* values were considered nominal and should be interpreted cautiously.

### 3.8. Adverse Events

No adverse events were reported or observed in either the EA or SA group during the treatment process.

## 4. Discussion

### 4.1. Clinical Efficacy and Cumulative Effects

In this randomized controlled trial, a six-session course of EA significantly alleviated pain intensity and disability in patients with CNLBP, accompanied by measurable changes in peripheral physiological responses. Between-group effect sizes for the primary clinical outcomes were generally large, with *r* values ranging from 0.64 to 0.84 for changes in NRS and ODI scores, suggesting substantial treatment-related differences in pain reduction and functional improvement. These large effect sizes were accompanied by clinically meaningful changes in most patients (MCID for NRS ≥ 2).

Responder analysis based on the MCID showed that although a considerable proportion of patients achieved clinically meaningful improvement after the first session, the responder rate was substantially higher after completion of the six-session EA course. This finding suggests a cumulative analgesic effect, in which repeated sessions may contribute to reaching clinically significant improvement. The concurrent improvement in activities of daily living further supports the functional relevance of this cumulative response. These results are consistent with previous evidence supporting the efficacy of EA for chronic pain [41,42] and provide a basis for further investigating its underlying peripheral physiological mechanisms.

### 4.2. EA-Induced Microcirculatory Responses

At the peripheral physiological level, we found that EA significantly increased local microcirculatory perfusion at the acupuncture site, which could be seen after each treatment and was sustained during the treatment course, while SA failed to alter the blood flow. This is consistent with the findings of earlier laser Doppler studies, which have found repetitive acupuncture stimulation to improve microcirculatory perfusion at acupoints and to be correlated with increased pain threshold [43]. Previous mechanistic research suggests that acupuncture may promote local vasodilation and influence sympathetic nerve activity by regulating the levels of vasoactive substances such as substance *p*, CGRP, and NO, thereby improving tissue perfusion [44,45]. In addition, acupuncture induces transient fluctuations in blood flow within neural tissue, and modulates endogenous pain regulatory networks [46,47]. Although enhanced lumbar microcirculation may alleviate muscle pain and facilitate tissue homeostasis at the periphery, whether this improved perfusion extends to deeper neural structures to promote neural recovery warrants further investigation [48,49]. These findings suggest that improved local microcirculation may represent a measurable peripheral physiological response associated with acupuncture-related analgesic effects.

### 4.3. Skin Temperature and Thermoregulatory Response

Skin temperature has been widely used as a noninvasive indicator of peripheral vascular activity and thermoregulatory responses [50,51,52]. In the present study, local skin temperature at BL23 increased significantly from baseline within the EA group after the first session (*r* = 0.68, *p* < 0.002). However, the between-group difference after the first session did not remain significant after Bonferroni adjustment (*r* = 0.26, raw *p* = 0.05, adjusted *p* = 0.10), and no significant between-group difference was observed after the sixth session (*r* = 0.17, *p* = 0.40). Therefore, this thermal change should be interpreted as a transient, session-sensitive within-group response rather than a confirmed treatment-specific between-group effect of EA. Although some previous studies have reported temperature increases accompanying pain relief [53], the present exploratory findings do not establish that EA specifically induces cutaneous vasodilation or thermoregulatory changes. Skin temperature may still be useful as an exploratory noninvasive physiological measure, but its role as a treatment-response marker remains uncertain and requires confirmation.

It is noted that the sham intervention, albeit non-penetrating, included skin contact and tactile stimulation at the same acupoints. This could have led to an activation of the cutaneous mechanoreceptors and local vasomotor response, which may have partially explained the changes in blood flow and skin temperature observed in the SA group. Therefore, the sham condition should be regarded as a physiological control with minimal sensory input rather than a completely inert intervention. Such baseline sensory activation may have attenuated the observed between-group differences in microcirculatory perfusion and skin temperature, meaning that the between-group effect sizes for microcirculatory outcomes should be interpreted as conservative estimates of the peripheral effects of EA.

### 4.4. Tissue Mechanical Properties

There were no consistent differences between groups in tissue mechanical properties (tone, stiffness, and elasticity) measured with MyotonPRO, despite significant differences in EA-induced effects on pain, function and peripheral physiological parameters. This negative result does not exclude potential alterations in deeper lumbar muscles since the device is mainly designed to measure superficial tissues (1–5 mm), while the muscles for electroacupuncture were 29–52 mm deep [54]. The lack of changes detected may also be due to the fact that the effects of electroacupuncture are likely to be neuromuscular modulation, local perfusion, and metabolic regulation rather than mechanical changes. Furthermore, these two weeks of treatment may have been insufficient to induce detectable changes in passive muscle biomechanics, and longer intervention may be necessary. Future studies may combine MyotonPRO with depth-sensitive techniques, such as ultrasound shear-wave elastography or advanced musculoskeletal imaging, to evaluate mechanical adaptations occurring in deeper muscles and fascial structures following EA.

### 4.5. Association Between Peripheral Physiological Responses and Clinical Improvement

In this study, local vascular and thermoregulatory responses to EA were examined in relation to pain and functional outcomes. Findings indicated that EA was accompanied by peripheral physiological changes, particularly increases in local blood flow, which occurred in parallel with reductions in pain and improvements in function. The effect sizes for changes in local blood flow ranged from moderate to large (r = 0.37–0.68), and changes in skin temperature exhibited a similar directional trend but with less consistent effect sizes.

Exploratory correlation analysis also revealed that increases in blood flow at BL23 were positively correlated with decreases in NRS scores, suggesting that local microcirculatory changes may be a potential physiological correlate of pain reduction. In contrast, this correlation between blood flow and ODI improvement was not significant. Skin temperature exhibited a modest correlation with ODI changes and a comparable but non-significant pattern with NRS decreases, suggesting that changes in skin temperature might be more transient and session-dependent than changes in blood flow. However, this analysis pooled participants from both treatment groups; therefore, the association may partly reflect differences between the groups and does not establish a relationship between physiological and clinical changes within either group. This finding should be regarded as exploratory and hypothesis-generating.

These physiological changes were observed at the predetermined principal acupoint BL23 as well as in the pain-sensitive area adjacent to the CNLBP region, suggesting that EA may be associated with localized peripheral physiological responses in pain-sensitive lumbar tissues. These changes in pain perception and local physiological responses may not be solely due to EA-specific effects, but result from several other factors, such as treatment context, patient expectations, and nonspecific autonomic regulation. Thus, local microcirculatory perfusion and skin temperature may serve as exploratory indicators of peripheral physiological responses to acupuncture stimulation. Further confirmatory research with standardized methods is still needed to ascertain whether local blood flow or skin temperature can be reliable indicators of clinical response.

### 4.6. Limitations

This study has several limitations. First, This was a single-center study with relatively restrictive eligibility criteria, including male participants aged 18–40 years, BMI 18.5–23.9 kg/m^2^, and predominantly left-sided unilateral low back pain. Although these criteria reduced heterogeneity and improved the consistency of local tissue measurements, they may limit the generalizability of our findings to older adults, overweight or obese individuals, or patients with more complex pain presentations. Furthermore, although microcirculatory perfusion and thermoregulatory responses were assessed, this exploratory study was not designed to establish these parameters as validated biomarkers of acupuncture efficacy. Therefore, the observed physiological responses should be interpreted as preliminary indicators of EA-induced peripheral adaptations, requiring confirmation in larger multicenter studies with more diverse populations.

Second, because of the inherent characteristics of acupuncture interventions, the acupuncturist could not be blinded to group allocation. Although participants and outcome assessors were blinded, and the acupuncturist had no access to outcome assessment or data entry, the possibility of performance bias cannot be completely excluded. In addition, blinding may have been incomplete. Although treatment-guessing did not differ significantly between groups (*p* = 0.11), successful blinding cannot be confirmed given the relatively small sample size. Although nonpenetrating blunt-tip needles were used to improve participant blinding and minimize specific needling effects, skin contact and stimulation at the same acupoints may still induce peripheral sensory input and nonspecific vascular or autonomic responses. Future studies should further optimize sham procedures, standardize practitioner–patient interactions, and incorporate objective assessments of autonomic regulation to better distinguish specific vascular responses induced by acupuncture.

Third, the responder definition was based on an absolute NRS reduction threshold, which may be influenced by baseline pain severity. Given the relatively modest baseline pain intensity in our sample, responder rates should be interpreted cautiously, and future studies may consider combining absolute and relative improvement thresholds.

## 5. Conclusions

EA improved pain and functional limitations in patients with CNLBP, with cumulative effects observed over the treatment course and maintained at the 4-week post-treatment follow-up. Enhanced local microcirculatory perfusion was accompanied by clinical improvement and correlated with pain reduction in a pooled exploratory analysis. However, this potential physiological correlate requires confirmation in future group-specific studies. Skin temperature changes were transient and did not show a confirmed treatment-specific between-group effect. Similar directional physiological responses were observed at the predefined principal acupoint, suggesting localized vascular and tissue adaptations associated with EA stimulation. These findings provide preliminary evidence supporting the use of objective peripheral physiological assessments for investigating acupuncture-related responses. Further studies are required to determine whether these physiological parameters can serve as reliable indicators of clinical response.

## Figures and Tables

**Table 1:** Demographics and Baseline Characteristics.

Characteristic	Group		
	EA (n = 30)	SA (n = 30)	*p* Value
Age, median (IQR), y	23.50 (21.00, 28.00)	24.00 (23.00, 26.00)	0.32
Weight, mean (SD), kg	66.47 (4.32)	67.58 (4.37)	0.33
Height, median (IQR), cm	170.75 (168.13, 174.50)	173.50 (169.75, 178.13)	0.18
Body mass index, median (IQR)	22.55 (21.88, 23.23)	22.35 (21.80, 23.33)	0.75
Education, No. (%)			0.22
Illiterate	0 (0.00)	0 (0.00)	
High school or below	21 (70.00)	25 (83.33)	
College or above	9 (30.00)	5 (16.67)	
Sitting time, median (IQR), h	4.00 (3.00, 5.63)	5.00 (4.00, 5.00)	0.22
Weekly exercise, median (IQR), h	6.00 (6.00, 9.00)	7.00 (6.00, 10.00)	0.35
Standing time, median (IQR), h	4.75 (4.00, 5.25)	5.00 (3.00, 5.25)	0.84
Low-back pain duration, median (IQR), month	24.00 (12.00, 39.00)	24.00 (16.50, 36.00)	0.75

Baseline characteristics are presented for all randomized participants (EA, n = 30; SA, n = 30). Data are presented as median (IQR), mean (SD), or number (%), as appropriate. *p* values are shown for descriptive purposes only; no formal hypothesis testing was performed for baseline comparisons. For descriptive purposes, *p* values were calculated using the Mann–Whitney U test for non-normally distributed continuous variables, independent-samples *t* test for normally distributed variables, and the χ^2^ test or Fisher’s exact test for categorical variables. No threshold for statistical significance was applied to these descriptive comparisons. EA: electroacupuncture; SA: sham electroacupuncture; IQR: interquartile range; SD: standard deviation.

**Table 2:** Comparison of Numeric Rating Scale scores in patients at different time points before and after treatment.

Time Point	Group	Median (Q1, Q3)	*r* (Within)	Raw *p* (Within)	Bonferroni-Adjusted *p* (Within)	Δ Median (Q1, Q3)	*r* (Between)	Raw *p* (Between)	Bonferroni-Adjusted *p* (Between)
Baseline	EA (n = 29)	5.00 (4.00, 5.00)	—	—	—	—	0.08	0.55	—
	SA (n = 30)	5.00 (4.00, 5.00)	—	—	—	—			
After 1st	EA (n = 29)	3.00 (3.00, 4.00)	0.89	<0.001	<0.003	−1.00 (−2.00, −1.00)	0.84	<0.001	<0.003
	SA (n = 30)	5.00 (4.00, 5.00)	0.32	0.08	0.24	0.00 (0.00, 0.00)			
After 6th	EA (n = 29)	0.00 (0.00, 1.00)	0.89	<0.001	<0.003	−4.00 (−5.00, −4.00)	0.78	<0.001	<0.003
	SA (n = 30)	4.00 (3.00, 5.00)	0.59	0.001	0.003	−0.50 (−2.00, 0.00)			
4-week follow-up	EA (n = 29)	1.00 (1.00, 2.00)	0.88	<0.001	<0.003	−3.00 (−4.00, −3.00)	0.78	<0.001	<0.003
	SA (n = 30)	5.00 (4.00, 6.00)	0.01	0.95	1.00	0.00 (−1.00, 1.00)			

Data are presented as median (Q1, Q3). Within-group comparisons were performed using the Wilcoxon signed-rank test, with each post-treatment time point compared with baseline. Between-group comparisons of change from baseline (Δ) were conducted using the Mann–Whitney U test. Effect size is expressed as *r* based on standardized test statistics. Raw *p* values and Bonferroni-adjusted *p* values are reported. Bonferroni correction was applied to multiple post-baseline comparisons, whereas baseline between-group comparisons were considered descriptive and were not included in the adjustment. All tests were two-sided, and adjusted *p* < 0.05 was considered statistically significant. EA: electroacupuncture; SA: sham electroacupuncture; Q1: first quartile; Q3: third quartile; Δ: change from baseline; NRS: Numeric Rating Scale.

**Table 3:** Comparison of the minimal clinically important difference in Numeric Rating Scale scores across different treatment time points.

Time Point	Group	ΔNRS ≥ 2, n (%)	ΔNRS < 2, n (%)	RR (95% CI)	Raw *p* Value	Bonferroni-Adjusted *p*
After 1st	EA (n = 29)	11 (37.93%)	18 (62.07%)	—	<0.001	<0.003
	SA (n = 30)	0 (0.00%)	30 (100.00%)			
After 6th	EA (n = 29)	29 (100.00%)	0 (0.00%)	3.75 (2.07–6.79)	<0.001	< 0.003
	SA (n = 30)	8 (26.67%)	22 (73.33%)			
4-week follow-up	EA (n = 29)	29 (100.00%)	0 (0.00%)	6.00 (2.70–13.35)	<0.001	<0.003
	SA (n = 30)	5 (16.67%)	25 (83.33%)			

The minimal clinically important difference (MCID) was defined as a reduction of ≥ 2 points on the NRS. Data are presented as number (%). Risk ratios (RRs) with 95% confidence intervals (CIs) were calculated from responder proportions. Group differences in the proportion of patients achieving the MCID were assessed using the χ^2^ test or Fisher’s exact test, as appropriate. Raw *p* values and Bonferroni-adjusted *p* values are reported. Bonferroni correction was applied to multiple post-baseline comparisons. All tests were two-sided, and adjusted *p* < 0.05 was considered statistically significant. EA: electroacupuncture; SA: sham electroacupuncture; NRS: Numeric Rating Scale; MCID: minimal clinically important difference; RR: risk ratio; CI: confidence interval.

**Table 4:** Comparison of Oswestry Disability Index scores in patients at different time points before and after treatment.

Time Point	Group	Median (Q1, Q3)	*r* (Within)	Raw *p* (Within)	Bonferroni-Adjusted *p* (Within)	Δ Median (Q1, Q3)	*r* (Between)	Raw *p* (Between)	Bonferroni-Adjusted *p* (Between)
Baseline	EA (n = 29)	12.00 (9.00, 14.00)	—	—	—	—	0.02	0.86	—
	SA (n = 30)	11.00 (11.00, 13.00)	—	—	—	—			
After 1st	EA (n = 29)	8.00 (7.00, 10.00)	0.88	<0.001	<0.002	−3.00 (−4.00, −2.00)	0.72	<0.001	<0.002
	SA (n = 30)	11.00 (9.00, 13.00)	0.44	0.02	0.04	−1.00 (−1.00, 1.00)			
After 6th	EA (n = 29)	3.00 (2.00, 4.00)	0.87	<0.001	<0.002	−9.00 (−11.00, −7.00)	0.64	<0.001	<0.002
	SA (n = 30)	9.00 (5.00, 12.00)	0.64	<0.001	<0.002	−3.00 (−5.00, 0.00)			

Data are presented as median (Q1, Q3). Within-group comparisons were performed using the Wilcoxon signed-rank test, with each post-treatment time point compared with baseline. Between-group comparisons of change from baseline (Δ) were conducted using the Mann–Whitney U test. Effect size is expressed as *r* based on standardized test statistics. Raw *p* values and Bonferroni-adjusted *p* values are reported. Bonferroni correction was applied to multiple post-baseline comparisons, whereas baseline between-group comparisons were considered descriptive and were not included in the adjustment. All tests were two-sided, and adjusted *p* < 0.05 was considered statistically significant. EA: electroacupuncture; SA: sham electroacupuncture; Q1: first quartile; Q3: third quartile; Δ: change from baseline; ODI: Oswestry Disability Index.

**Table 5:** Comparison of microcirculatory perfusion at the Shenshu (BL23) acupoint at different time points before and after treatment.

Time Point	Group	Median (Q1, Q3)	*r* (Within)	Raw *p* (Within)	Bonferroni-Adjusted *p* (Within)	Δ Median (Q1, Q3)	*r* (Between)	Raw *p* (Between)	Bonferroni-Adjusted *p* (Between)
Baseline	EA (n = 29)	225.32 (205.17, 253.65)	—	—	—	—	0.14	0.28	—
	SA (n = 30)	216.40 (197.56, 247.30)	—	—	—	—			
After 1st	EA (n = 29)	257.74 (226.64, 306.09)	0.68	0.001	<0.002	37.85 (6.67, 63.14)	0.37	0.004	0.008
	SA (n = 30)	214.30 (191.93, 248.07)	0.04	0.81	1.00	−9.51 (−18.74, 25.61)			
After 6th	EA (n = 29)	269.47 (244.66, 315.35)	0.64	0.001	0.002	38.15 (8.13, 84.51)	0.40	0.002	0.004
	SA (n = 30)	223.01 (206.48, 232.57)	0.03	0.88	1.00	−1.81 (−26.69, 33.65)			

Data are presented as median (Q1, Q3). microcirculatory perfusion was measured using a laser speckle contrast imaging system. Within-group comparisons were performed using the Wilcoxon signed-rank test, with each post-treatment time point compared with baseline. Between-group comparisons of change from baseline (Δ) were conducted using the Mann–Whitney U test. Effect size is expressed as r based on standardized test statistics. Raw *p* values and Bonferroni-adjusted *p* values are reported. Bonferroni correction was applied to multiple post-baseline comparisons, whereas baseline between-group comparisons were considered descriptive and were not included in the adjustment. All tests were two-sided, and adjusted *p* < 0.05 was considered statistically significant. EA: electroacupuncture; SA: sham electroacupuncture; Q1: first quartile; Q3: third quartile; Δ: change from baseline.

**Table 6:** Comparison of skin temperature at the Shenshu (BL23) at different time points before and after treatment.

Time Point	Group	Median (Q1, Q3)	*r* (within)	Raw *p* (Within)	Bonferroni-Adjusted *p* (Within)	Δ Median (Q1, Q3)	*r* (Between)	Raw *p* (Between)	Bonferroni-Adjusted *p* (Between)
Baseline	EA (n = 29)	33.06 (32.10, 33.65)	—	—	—	—	0.03	0.82	—
	SA (n = 30)	33.13 (32.50, 33.71)	—	—	—	—			
After 1st	EA (n = 29)	33.51 (33.21, 33.99)	0.68	<0.001	<0.002	0.45 (0.13, 0.91)	0.26	0.05	0.10
	SA (n = 30)	33.40 (32.87, 33.77)	0.33	0.07	0.14	0.08 (−0.16, 0.76)			
After 6th	EA (n = 29)	33.37 (32.98, 33.85)	0.33	0.07	0.14	0.51 (−0.41, 1.16)	0.17	0.20	0.40
	SA (n = 30)	33.02 (32.62, 33.79)	0.12	0.52	1.00	0.16 (−0.42, 0.67)			

Data are presented as median (Q1, Q3). Skin temperature was measured using infrared thermography. Within-group comparisons were performed using the Wilcoxon signed-rank test, with each post-treatment time point compared with baseline. Between-group comparisons of change from baseline (Δ) were conducted using the Mann–Whitney U test. Effect size is expressed as *r* based on standardized test statistics. Raw *p* values and Bonferroni-adjusted *p* values are reported. Bonferroni correction was applied to multiple post-baseline comparisons, whereas baseline between-group comparisons were considered descriptive and were not included in the adjustment. All tests were two-sided, and adjusted *p* < 0.05 was considered statistically significant. EA: electroacupuncture; SA: sham electroacupuncture; Q1: first quartile; Q3: third quartile; Δ: change from baseline.

**Table 7:** Comparison of muscle biomechanical indices at the Shenshu (BL23) acupoint between the EA and SA groups at different time points before and after treatment.

Index	Time Point	Group	Median (Q1, Q3)	*r* (Within)	Raw *p* (Within)	Bonferroni-Adjusted *p* (Within)	Δ Median (Q1, Q3)	*r* (Between)	Raw *p* (Between)	Bonferroni-Adjusted *p* (Between)
F	Baseline	EA (n = 29)	14.60 (14.20, 15.40)	—	—	—	—	0.01	0.92	—
		SA (n = 30)	14.45 (14.00, 15.50)	—	—	—	—			
	After 1st	EA (n = 29)	15.10 (14.50, 15.80)	0.70	<0.001	<0.002	0.40 (0.10, 0.70)	0.08	0.55	1.00
		SA (n = 30)	14.95 (14.30, 15.70)	0.44	0.02	0.04	0.30 (−0.20, 0.70)			
	After 6th	EA (n = 29)	14.50 (14.00, 15.50)	0.11	0.56	1.00	0.10 (−0.30, 0.60)	0.02	0.90	1.00
		SA (n = 30)	14.50 (14.10, 15.20)	0.06	0.74	1.00	0.10 (−0.40, 0.70)			
S	Baseline	EA (n = 29)	264.00 (235.00, 284.00)	—	—	—	—	0.03	0.84	—
		SA (n = 30)	251.00 (230.00, 282.00)	—	—	—	—			
	After 1st	EA (n = 29)	287.00 (246.00, 293.00)	0.79	<0.001	<0.002	21.00 (16.00, 36.00)	0.16	0.23	0.46
		SA (n = 30)	265.00 (238.00, 303.00)	0.58	0.001	0.002	21.50 (3.00, 33.00)			
	After 6th	EA (n = 29)	238.00 (220.00, 285.00)	0.16	0.39	0.78	−4.00 (−20.00, 17.00)	0.05	0.72	1.00
		SA (n = 30)	256.00 (211.00, 295.00)	0.15	0.41	0.82	−3.50 (−37.00, 23.00)			
D	Baseline	EA (n = 29)	0.88 (0.80, 0.95)	—	—	—	—	0.10	0.42	—
		SA (n = 30)	0.89 (0.83, 1.00)	—	—	—	—			
	After 1st	EA (n = 29)	0.86 (0.80, 0.98)	0.35	0.06	0.12	0.04 (0.00, 0.09)	0.15	0.25	0.50
		SA (n = 30)	0.90 (0.83, 1.02)	0.19	0.30	0.60	0.02 (−0.02, 0.05)			
	After 6th	EA (n = 29)	0.85 (0.75, 0.90)	0.28	0.14	0.28	−0.03 (−0.18, 0.06)	0.07	0.58	1.00
		SA (n = 30)	0.92 (0.77, 0.99)	0.15	0.40	0.80	−0.01 (−0.15, 0.10)			

Data are presented as median (Q1, Q3). Within-group comparisons were performed using the Wilcoxon signed-rank test, with each post-treatment time point compared with baseline. Between-group comparisons of change from baseline (Δ) were conducted using the Mann–Whitney U test. Effect size is expressed as *r* based on standardized test statistics. Raw *p* values and Bonferroni-adjusted *p* values are reported. Bonferroni correction was applied to multiple post-baseline comparisons, whereas baseline between-group comparisons were considered descriptive and were not included in the adjustment. All tests were two-sided, and adjusted *p* < 0.05 was considered statistically significant. EA: electroacupuncture; SA: sham electroacupuncture; Q1: first quartile; Q3: third quartile; Δ: change from baseline; F: muscle tone; S: muscle stiffness; D: muscle elasticity.

**Table 8:** Exploratory correlations between changes in local physiological parameters and clinical outcomes.

Δ Physiological Parameter	Δ Clinical Outcome	Spearman’s ρ (95% CI)	*p* Value
Δ Blood flow at BL23	Δ NRS	−0.34 (−0.55, −0.09)	0.008
	Δ ODI	−0.20 (−0.43, 0.06)	0.14
Δ Skin temperature at BL23	Δ NRS	−0.23 (−0.46, 0.03)	0.08
	Δ ODI	−0.27 (−0.49, −0.01)	0.04

Spearman correlations were calculated between changes in local physiological parameters and changes in clinical outcomes from baseline to after the sixth session in the combined sample (n = 59). Δ values were calculated as post-treatment values minus baseline values. Spearman’s rank correlation coefficients (ρ) and 95% confidence intervals (CIs), estimated using Fisher’s z transformation, are reported. Negative ρ values indicate that increases in physiological parameters were associated with decreases in NRS or ODI scores, reflecting clinical improvement. NRS: Numeric Rating Scale; ODI: Oswestry Disability Index; CI: confidence interval.

## Data Availability

The data presented in this study are available on request from the corresponding author due to ethical restrictions related to participant privacy and the protection of personal information.

## Supplementary Materials

The following supporting information can be downloaded at: https://www.mdpi.com/article/10.3390/healthcare14213792/s1, Figure S1; Table S1–S12.
