# Supplementary material for: Peripheral Vascular and Physiological Responses Associated with Electroacupuncture in Chronic Nonspecific Low Back Pain: A Randomized Controlled Trial
Source: Healthcare (Basel). 2026 Nov 6;14(21):3792. doi: 10.3390/healthcare14213792 (PMC13763537; doi:10.3390/healthcare14213792)
Supplement: Supplementary file 1 [file healthcare-14-03792-s001.zip › healthcare-4584996-supplementary.pdf]

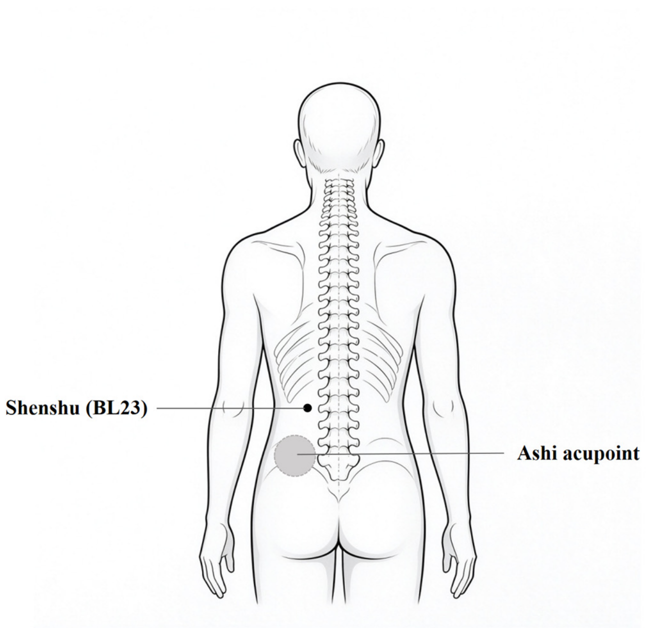


**Figure S1. The figure shows the locations of Shenshu (BL23) and the Ashi acupoint on the lower back.** Shenshu (BL23) is located 1.5 cun lateral to the lower border of the spinous process of the second lumbar vertebra (L2). The Ashi acupoint was defined as a pain-sensitive site within the L5/S1 segment, identified by palpation. The gray shaded area indicates the region of the Ashi acupoint. BL23: Shenshu; L2: second lumbar vertebra; L5/S1: fifth lumbar/first sacral segment.

**Table S1. Microcirculatory perfusion at acupuncture sites at different time points**

| Time point | Group | Shenshu (BL23) | Ipsilateral Ashi acupoint |
|---|---|---|---|
| Baseline | EA (n = 29) | 232.21 ± 42.05 | 218.42 ± 38.30 |
| | SA (n = 30) | 220.53 ± 29.45 | 213.82 ± 29.51 |
| After 1st | EA (n = 29) | 269.13 ± 61.44 | 247.36 ± 61.39 |
| | SA (n = 30) | 225.22 ± 44.71 | 214.28 ± 45.94 |
| After 6th | EA (n = 29) | 280.55 ± 64.92 | 254.15 ± 48.01 |
| | SA (n = 30) | 222.03 ± 31.10 | 204.93 ± 29.18 |

Data are presented as mean ± SD. Microcirculatory perfusion was measured at the Shenshu (BL23) and ipsilateral Ashi acupoints at baseline, after the first treatment session, and after the sixth session. EA: electroacupuncture; SA: sham electroacupuncture; SD: standard deviation.

**Table S2. Comparison of microcirculatory perfusion at ipsilateral Ashi acupoints within the L5/S1 segment before and after treatment**

| Time point | Group | Median (Q1, Q3) | *r* (within) | Raw *P* (within) | Bonferroni-adjusted *P* (within) | Δ Median (Q1, Q3) | *r* (between) | Raw *P* (between) | Bonferroni-adjusted *P* (between) |
|---|---|---|---|---|---|---|---|---|---|
| Baseline | EA (n = 29) | 215.66 (193.99, 243.98) | — | — | | — | 0.08 | 0.54 | — |
| | SA (n = 30) | 209.87 (192.16, 237.40) | — | — | | — | | | |
| After 1st | EA (n = 29) | 246.71 (199.94, 264.50) | 0.47 | 0.01 | 0.02 | 21.88 (−2.78, 53.18) | 0.31 | 0.02 | 0.04 |
| | SA (n = 30) | 209.47 (180.18, 231.08) | 0.13 | 0.49 | 0.98 | −7.34 (−28.39, 25.28) | | | |
| After 6th | EA (n = 29) | 254.06 (225.64, 277.48) | 0.69 | < 0.001 | < 0.002 | 20.45 (7.30, 56.43) | 0.50 | 0.001 | 0.002 |
| | SA (n = 30) | 202.74 (182.00, 224.47) | 0.28 | 0.12 | 0.24 | −14.18 (−37.19, 13.61) | | | |

Data are presented as median (Q1, Q3). Within-group comparisons were performed using the Wilcoxon signed-rank test, with each post-treatment time point compared with baseline. Between-group comparisons of change from baseline (Δ) were conducted using the Mann–Whitney U test. Effect size is expressed as *r* based on standardized test statistics. Raw *P* values and Bonferroni-adjusted *P* values are reported. Bonferroni correction was applied to multiple post-baseline comparisons, whereas baseline between-group comparisons were considered descriptive and were not included in the adjustment. All tests were two-sided, and adjusted $P < 0.05$ was considered statistically significant. EA: electroacupuncture; SA: sham electroacupuncture; Q1: first quartile; Q3: third quartile; Δ: change from baseline.

**Table S3. Skin temperature at acupuncture sites at different time points**

| Time point | Group | Shenshu (BL23) | Ipsilateral Ashi acupoint |
|---|---|---|---|
| Baseline | EA (n = 29) | 32.93 ± 0.97 | 33.28 ± 0.84 |
| | SA (n = 30) | 33.01 ± 0.90 | 33.30 ± 0.92 |
| After 1st | EA (n = 29) | 33.51 ± 0.57 | 33.55 ± 0.55 |
| | SA (n = 30) | 33.24 ± 0.74 | 33.28 ± 0.96 |
| After 6th | EA (n = 29) | 33.35 ± 0.61 | 33.32 ± 0.72 |
| | SA (n = 30) | 33.01 ± 0.78 | 33.27 ± 0.90 |

Data are presented as mean ± SD. Skin temperature at the Shenshu (BL23) and ipsilateral Ashi acupoints was measured at baseline, after the first treatment session, and after the sixth session. EA: electroacupuncture; SA: sham electroacupuncture; SD: standard deviation.

**Table S4. Comparison of skin temperature at ipsilateral Ashi acupoint within the L5/S1 segment at different time points before and after treatment**

| Time point | Group | Median (Q1, Q3) | *r* (within) | Raw *P* (within) | Bonferroni-adjusted *P* (within) | Δ Median (Q1, Q3) | *r* (between) | Raw *P* (between) | Bonferroni-adjusted *P* (between) |
|---|---|---|---|---|---|---|---|---|---|
| Baseline | EA (n = 29) | 33.27 (32.85, 33.92) | — | — | — | — | 0.01 | 0.92 | — |
| | SA (n = 30) | 33.24 (32.88, 34.12) | — | — | — | — | | | |
| After 1st | EA (n = 29) | 33.55 (33.19, 33.90) | 0.37 | 0.05 | 0.10 | 0.18 (−0.08, 0.70) | 0.20 | 0.12 | 0.24 |
| | SA (n = 30) | 33.49 (32.80, 33.88) | 0.02 | 0.89 | 1.00 | 0.06 (−0.35, 0.40) | | | |
| After 6th | EA (n = 29) | 33.35 (32.76, 33.87) | 0.10 | 0.60 | 1.00 | 0.24 (−0.74, 0.63) | 0.09 | 0.48 | 0.96 |
| | SA (n = 30) | 33.13 (32.79, 34.07) | 0.05 | 0.77 | 1.00 | −0.07 (−0.62, 0.62) | | | |

Data are presented as median (Q1, Q3). Within-group comparisons were performed using the Wilcoxon signed-rank test, with each post-treatment time point compared with baseline. Between-group comparisons of change from baseline (Δ) were conducted using the Mann–Whitney U test. Effect size is expressed as *r* based on standardized test statistics. Raw *P* values and Bonferroni-adjusted *P* values are reported. Bonferroni correction was applied to multiple post-baseline comparisons, whereas baseline between-group comparisons were considered descriptive and were not included in the adjustment. All tests were two-sided, and adjusted $P < 0.05$ was considered statistically significant. EA: electroacupuncture; SA: sham electroacupuncture; Q1: first quartile; Q3: third quartile; Δ: change from baseline.

**Table S5. MyotonPRO-derived muscle biomechanical properties before and after treatment**

| Time point | Group | Shenshu (BL23) | | | Ipsilateral Ashi acupoint | | |
|---|---|---|---|---|---|---|---|
| | | Muscle tone (F) | Muscle stiffness (S) | Muscle elasticity (D) | Muscle tone (F) | Muscle stiffness (S) | Muscle elasticity (D) |
| Baseline | EA (n = 29) | 14.72 ± 0.99 | 260.21 ± 39.98 | 0.88 ± 0.15 | 15.86 ± 1.58 | 309.90 ± 69.19 | 0.90 ± 0.11 |
| | SA (n = 30) | 14.69 ± 1.08 | 260.70 ± 49.18 | 0.92 ± 0.19 | 15.80 ± 1.81 | 301.73 ± 70.97 | 0.90 ± 0.13 |
| After 1st | EA (n = 29) | 15.09 ± 0.90 | 282.59 ± 43.66 | 0.92 ± 0.18 | 16.31 ± 1.58 | 329.21 ± 67.46 | 0.95 ± 0.09 |
| | SA (n = 30) | 14.95 ± 1.09 | 275.33 ± 54.34 | 0.92 ± 0.19 | 16.21 ± 2.24 | 316.77 ± 80.01 | 0.94 ± 0.15 |
| After 6th | EA (n = 29) | 14.76 ± 1.03 | 252.14 ± 42.73 | 0.83 ± 0.13 | 15.79 ± 1.37 | 304.90 ± 58.24 | 0.86 ± 0.12 |
| | SA (n = 30) | 14.70 ± 1.08 | 255.17 ± 46.95 | 0.89 ± 0.13 | 15.57 ± 1.84 | 290.97 ± 75.46 | 0.87 ± 0.14 |

Data are presented as mean ± standard deviation (SD). Muscle biomechanical properties, including muscle tone (F), muscle stiffness (S), and muscle elasticity (D), were assessed at the Shenshu (BL23) and ipsilateral Ashi acupoints using a handheld MyotonPRO device at baseline, after the first treatment session, and after the sixth session. EA: electroacupuncture; SA: sham electroacupuncture; SD: standard deviation; F: muscle tone; S: muscle stiffness; D: muscle elasticity.

**Table S6. Comparison of muscle biomechanical indices at ipsilateral Ashi acupoints within the L5/S1 segment between the EA and sham groups at different time points before and after treatment**

| Index | Time point | Group | Median (Q1, Q3) | *r* (within) | Raw *P* (within) | Bonferroni-adjusted *P* (within) | Δ Median (Q1, Q3) | *r* (between) | Raw *P* (between) | Bonferroni-adjusted *P* (between) |
|---|---|---|---|---|---|---|---|---|---|---|
| F | Baseline | EA (n = 29) | 15.80 (14.80, 16.70) | — | — | — | — | 0.07 | 0.60 | — |
| | | SA (n = 30) | 15.50 (14.80, 16.50) | — | — | — | — | | | |
| | After 1st | EA (n = 29) | 16.20 (15.20, 17.20) | 0.67 | < 0.001 | < 0.002 | 0.40 (0.30, 0.60) | 0.08 | 0.53 | 1.00 |
| | | SA (n = 30) | 15.90 (15.00, 17.20) | 0.49 | 0.008 | 0.02 | 0.35 (−0.10, 0.70) | | | |
| | After 6th | EA (n = 29) | 15.80 (15.00, 17.00) | 0.10 | 0.58 | 1.00 | −0.10 (−0.50, 0.30) | 0.16 | 0.22 | 0.44 |
| | | SA (n = 30) | 15.00 (14.40, 16.10) | 0.34 | 0.07 | 0.14 | −0.30 (−1.00, 0.10) | | | |
| S | Baseline | EA (n = 29) | 309.00 (270.00, 345.00) | — | — | — | — | 0.13 | 0.32 | — |
| | | SA (n = 30) | 290.50 (267.00, 327.00) | — | — | — | — | | | |
| | After 1st | EA (n = 29) | 331.00 (292.00, 360.00) | 0.66 | < 0.001 | < 0.002 | 21.00 (2.00, 40.00) | 0.06 | 0.66 | 1.00 |
| | | SA (n = 30) | 304.50 (270.00, 359.00) | 0.43 | 0.02 | 0.04 | 11.00 (−5.00, 48.00) | | | |
| | After 6th | EA (n = 29) | 304.00 (248.00, 360.00) | 0.10 | 0.60 | 1.00 | −4.00 (−27.00, 22.00) | 0.11 | 0.38 | 0.76 |
| | | SA (n = 30) | 279.00 (236.00, 341.00) | 0.28 | 0.12 | 0.24 | −11.00 (−36.00, 4.00) | | | |
| D | Baseline | EA (n = 29) | 0.89 (0.85, 0.92) | — | — | — | — | 0.01 | 0.93 | — |
| | | SA (n = 30) | 0.89 (0.81, 0.99) | — | — | — | — | | | |
| | After 1st | EA (n = 29) | 0.94 (0.89, 0.99) | 0.69 | 0.001 | 0.002 | 0.05 (0.03, 0.09) | 0.21 | 0.11 | 0.22 |
| | | SA (n = 30) | 0.92 (0.88, 0.98) | 0.46 | 0.01 | 0.02 | 0.02 (−0.01, 0.07) | | | |
| | After 6th | EA (n = 29) | 0.84 (0.80, 0.92) | 0.40 | 0.03 | 0.06 | −0.05 (−0.12, 0.01) | 0.12 | 0.36 | 0.72 |
| | | SA (n = 30) | 0.86 (0.79, 0.92) | 0.21 | 0.26 | 0.52 | −0.02 (−0.08, 0.05) | | | |

Data are presented as median (Q1, Q3). Within-group comparisons were performed using the Wilcoxon signed-rank test, with each post-treatment time point compared with baseline. Between-group comparisons of change from baseline (Δ) were conducted using the Mann–Whitney U test. Effect size is expressed as *r* based on standardized test statistics. Raw P values and Bonferroni-adjusted P values are reported. Bonferroni correction was applied to multiple post-baseline comparisons, whereas baseline between-group comparisons were considered descriptive and were not included in the adjustment. All tests were two-sided, and adjusted $P < 0.05$ was considered statistically significant. EA: electroacupuncture; SA: sham electroacupuncture; Q1: first quartile; Q3: third quartile; Δ: change from baseline; F: muscle tone; S: muscle stiffness; D: muscle elasticity.

**Table S7. ITT sensitivity analysis for NRS**

| Time point | Group | Median (Q1, Q3) | *r* (within) | Raw *P* (within) | Bonferroni-adjusted *P* (within) | Δ Median (Q1, Q3) | *r* (between) | Raw *P* (between) | Bonferroni-adjusted *P* (between) |
|---|---|---|---|---|---|---|---|---|---|
| Baseline | EA (n = 30) | 5.00 (4.00, 5.00) | — | — | — | — | 0.06 | 0.65 | — |
| | SA (n = 30) | 5.00 (4.00, 5.00) | — | — | — | — | | | |
| After 1st | EA (n = 30) | 3.00 (3.00, 4.00) | 0.87 | < 0.001 | < 0.003 | −1.00 (−2.00, −1.00) | 0.81 | < 0.001 | < 0.003 |
| | SA (n = 30) | 5.00 (4.00, 5.00) | 0.32 | 0.08 | 0.24 | 0.00 (0.00, 0.00) | | | |
| After 6th | EA (n = 30) | 0.00 (0.00, 1.00) | 0.87 | < 0.001 | < 0.003 | −4.00 (−5.00, −3.00) | 0.74 | < 0.001 | < 0.003 |
| | SA (n = 30) | 4.00 (3.00, 5.00) | 0.59 | 0.001 | 0.003 | −0.50 (−2.00, 0.00) | | | |
| 4-week follow-up | EA (n = 30) | 1.00 (1.00, 2.00) | 0.87 | < 0.001 | < 0.003 | −3.00 (−4.00, −2.00) | 0.76 | < 0.001 | < 0.003 |
| | SA (n = 30) | 5.00 (4.00, 6.00) | 0.01 | 0.95 | 1.00 | 0.00 (−1.00, 1.00) | | | |

Data are presented as median (Q1, Q3). This table presents the intention-to-treat sensitivity analysis, including all randomized participants. Missing post-baseline NRS values for the participant who withdrew were imputed using baseline observation carried forward. Within-group comparisons were performed using the Wilcoxon signed-rank test, with each post-treatment time point compared with baseline. Between-group comparisons of change from baseline (Δ) were conducted using the Mann–Whitney U test. Effect size is expressed as *r* based on standardized test statistics. Raw *P* values and Bonferroni-adjusted *P* values are reported. Bonferroni correction was applied to multiple post-baseline comparisons, whereas baseline between-group comparisons were considered descriptive and were not included in the adjustment. All tests were two-sided, and adjusted $P < 0.05$ was considered statistically significant. EA: electroacupuncture; SA: sham electroacupuncture; Q1: first quartile; Q3: third quartile; Δ: change from baseline; NRS: Numeric Rating Scale; ITT: intention-to-treat.

**Table S8. ITT sensitivity analysis for MCID responder analysis**

| Time point | Group | ΔNRS ≥ 2, n (%) | ΔNRS < 2, n (%) | RR (95% CI) | Raw *P* | Bonferroni-adjusted *P* |
|---|---|---|---|---|---|---|
| After 1st | EA (n = 30) | 11 (36.67%) | 19 (63.33%) | — | < 0.001 | < 0.003 |
| | SA (n = 30) | 0 (0.00%) | 30 (100.00%) | | | |
| After 6th | EA (n = 30) | 29 (96.67%) | 1 (3.33%) | 3.63 (1.99–6.58) | < 0.001 | < 0.003 |
| | SA (n = 30) | 8 (26.67%) | 22 (73.33%) | | | |
| 4-week follow-up | EA (n = 30) | 29 (96.67%) | 1 (3.33%) | 5.80 (2.60–12.94) | < 0.001 | < 0.003 |
| | SA (n = 30) | 5 (16.67%) | 25 (83.33%) | | | |

The minimal clinically important difference (MCID) was defined as a reduction of ≥ 2 points on the Numeric Rating Scale. This table presents the intention-to-treat sensitivity analysis, including all randomized participants. Missing post-baseline NRS values for the participant who withdrew were imputed using baseline observation carried forward. Risk ratios (RRs) with 95% confidence intervals (CIs) were calculated from responder proportions. Group differences in the proportion of patients achieving the MCID were assessed using the $\chi^2$ test or Fisher's exact test, as appropriate. Raw *P* values and Bonferroni-adjusted *P* values are reported. Bonferroni correction was applied to multiple post-baseline comparisons. All tests were two-sided, and adjusted $P < 0.05$ was considered statistically significant. EA: electroacupuncture; SA: sham electroacupuncture; NRS: Numeric Rating Scale; MCID: minimal clinically important difference; RR: risk ratio; CI: confidence interval; ITT: intention-to-treat.

**Table S9. ITT sensitivity analysis for ODI**

| Time point | Group | Median (Q1, Q3) | *r* (within) | Raw *P* (within) | Bonferroni-adjusted *P* (within) | Δ Median (Q1, Q3) | *r* (between) | Raw *P* (between) | Bonferroni-adjusted *P* (between) |
|---|---|---|---|---|---|---|---|---|---|
| Baseline | EA (n = 30) | 12.00 (9.00, 14.00) | — | — | — | — | 0.01 | 0.96 | — |
| | SA (n = 30) | 11.00 (11.00, 13.00) | — | — | — | — | | | |
| After 1st | EA (n = 30) | 8.50 (7.00, 10.00) | 0.86 | < 0.001 | < 0.002 | −3.00 (−4.00, −2.00) | 0.68 | < 0.001 | < 0.002 |
| | SA (n = 30) | 11.00 (9.00, 13.00) | 0.44 | 0.02 | 0.04 | −1.00 (−1.00, 1.00) | | | |
| After 6th | EA (n = 30) | 3.00 (2.00, 4.00) | 0.86 | < 0.001 | < 0.002 | −9.00 (−11.00, −6.00) | 0.61 | < 0.001 | < 0.002 |
| | SA (n = 30) | 9.00 (5.00, 12.00) | 0.64 | < 0.001 | < 0.002 | −3.00 (−5.00, 0.00) | | | |

Data are presented as median (Q1, Q3). This table presents the intention-to-treat sensitivity analysis, including all randomized participants. Missing post-baseline ODI values for the participant who withdrew were imputed using baseline observation carried forward. Within-group comparisons were performed using the Wilcoxon signed-rank test, with each post-treatment time point compared with baseline. Between-group comparisons of change from baseline (Δ) were conducted using the Mann–Whitney U test. Effect size is expressed as *r* based on standardized test statistics. Raw *P* values and Bonferroni-adjusted *P* values are reported. Bonferroni correction was applied to multiple post-baseline comparisons, whereas baseline between-group comparisons were considered descriptive and were not included in the adjustment. All tests were two-sided, and adjusted $P < 0.05$ was considered statistically significant. EA: electroacupuncture; SA: sham electroacupuncture; Q1: first quartile; Q3: third quartile; Δ: change from baseline; ODI: Oswestry Disability Index; ITT: intention-to-treat.

**Table S10. Demographics and Baseline Characteristics**

| Characteristic | Group | |
|---|---|---|
| | EA (n = 30) | SA (n = 30) |
| Age, mean (SD), y | 24.10 (3.36) | 24.97 (3.00) |
| Weight, mean (SD), kg | 66.47 (4.32) | 67.58 (4.37) |
| Height, mean (SD), cm | 172.23 (5.98) | 173.68 (5.16) |
| Body mass index, mean (SD) | 22.42 (1.09) | 22.41 (1.01) |
| Education, No. (%) | | |
| Illiterate | 0 (0.00) | 0 (0.00) |
| High school or below | 21 (70.00) | 25 (83.33) |
| College or above | 9 (30.00) | 5 (16.67) |
| Sitting time, mean (SD), h | 4.35 (1.89) | 4.55 (1.07) |
| Weekly exercise, mean (SD), h | 7.60 (2.51) | 7.83 (2.47) |
| Standing time, mean (SD), h | 4.70 (1.21) | 4.70 (1.68) |
| Low-back pain duration, mean (SD), month | 30.70 (23.18) | 29.80 (16.98) |

Baseline characteristics are presented for all randomized participants (EA, n = 30; SA, n = 30). Data are presented as mean ± standard deviation (SD), or number (%), as appropriate. EA: electroacupuncture; SA: sham electroacupuncture; SD: standard deviation.

**Table S11. Numeric Rating Scale scores at acupuncture sites at different time points**

| Time point | Group | Mean (SD) |
|---|---|---|
| Baseline | EA (n = 29) | 4.83 (0.81) |
| | SA (n = 30) | 4.70 (0.75) |
| After 1st | EA (n = 29) | 3.41 (0.78) |
| | SA (n = 30) | 4.60 (0.72) |
| After 6th | EA (n = 29) | 0.66 (0.86) |
| | SA (n = 30) | 3.73 (1.55) |
| 4-week follow-up | EA (n = 29) | 1.41 (1.09) |
| | SA (n = 30) | 4.67 (1.52) |

Data are presented as mean ± standard deviation (SD). EA: electroacupuncture; SA: sham electroacupuncture; SD: standard deviation; NRS: Numeric Rating Scale.

**Table S12. Oswestry Disability Index scores at acupuncture sites at different time points**

| Time point | Group | Mean (SD) |
|---|---|---|
| Baseline | EA (n = 29) | 12.10 (3.05) |
| | SA (n = 30) | 11.93 (2.53) |
| After 1st | EA (n = 29) | 8.41 (1.97) |
| | SA (n = 30) | 11.33 (2.63) |
| After 6th | EA (n = 29) | 2.72 (1.46) |
| | SA (n = 30) | 8.77 (3.86) |

Data are presented as mean ± standard deviation (SD). EA: electroacupuncture; SA: sham electroacupuncture; SD: standard deviation; ODI: Oswestry Disability Index.
